# Supplementary material for: Photochemical Efficiency and Oxidative Metabolism of Tree Species during Acclimation to High and Low Irradiance
Source: Plants (Basel). 2020 Aug 17;9(8):1047. doi: 10.3390/plants9081047 (PMC7463730; doi:10.3390/plants9081047)
Supplement: Supplementary file 1 [file plants-09-01047-s001.pdf]

SUPLEMENTARY FILES

Table S1: ANOVA results for environments, species and interactions (environments x species) showing statistic F and p-value for chlorophylls *a, b* and total, carotenoids (mass and area basis), ratio chlorophyll a: chlorophyll b and ratio total chlorophylls:carotenoids.

**Variable**	**Environment**		**Specie**		**Interaction**	
	**F**	**p**	**F**	**p**	**F**	**p**
Chl *a* [µmol g^{-1} (FM)]	176.85	<0.001	14.14	<0.001	4.58	<0.001
Chl *b* [µmol g^{-1} (FM)]	250.79	<0.001	22.51	<0.001	16.47	<0.001
Chl $_{tot}$ [µmol g^{-1} (FM)]	207.46	<0.001	16.75	<0.001	7.45	<0.001
Car [µmol g^{-1} (FM)]	151.59	<0.001	21.52	<0.001	8.77	<0.001
Chl *a* (µmol m^{-2})	77.33	<0.001	4.44	0.002	6.86	<0.001
Chl *b* (µmol m^{-2})	117.58	<0.001	8.05	<0.001	10.31	<0.001
Chl $_{tot}$ (µmol m^{-2})	92.88	<0.001	5.43	<0.001	7.66	<0.001
Car (µmol m^{-2})	55.74	<0.001	7.18	<0.001	9.20	<0.001
Chl *a*:chl *b*	44.84	<0.001	3.37	0.011	2.66	0.013
Chl $_{tot}$: Car	80.82	<0.001	5.46	<0.001	1.96	0.064
SPAD	281.71	<0.001	20.54	<0.001	18.63	<0.001

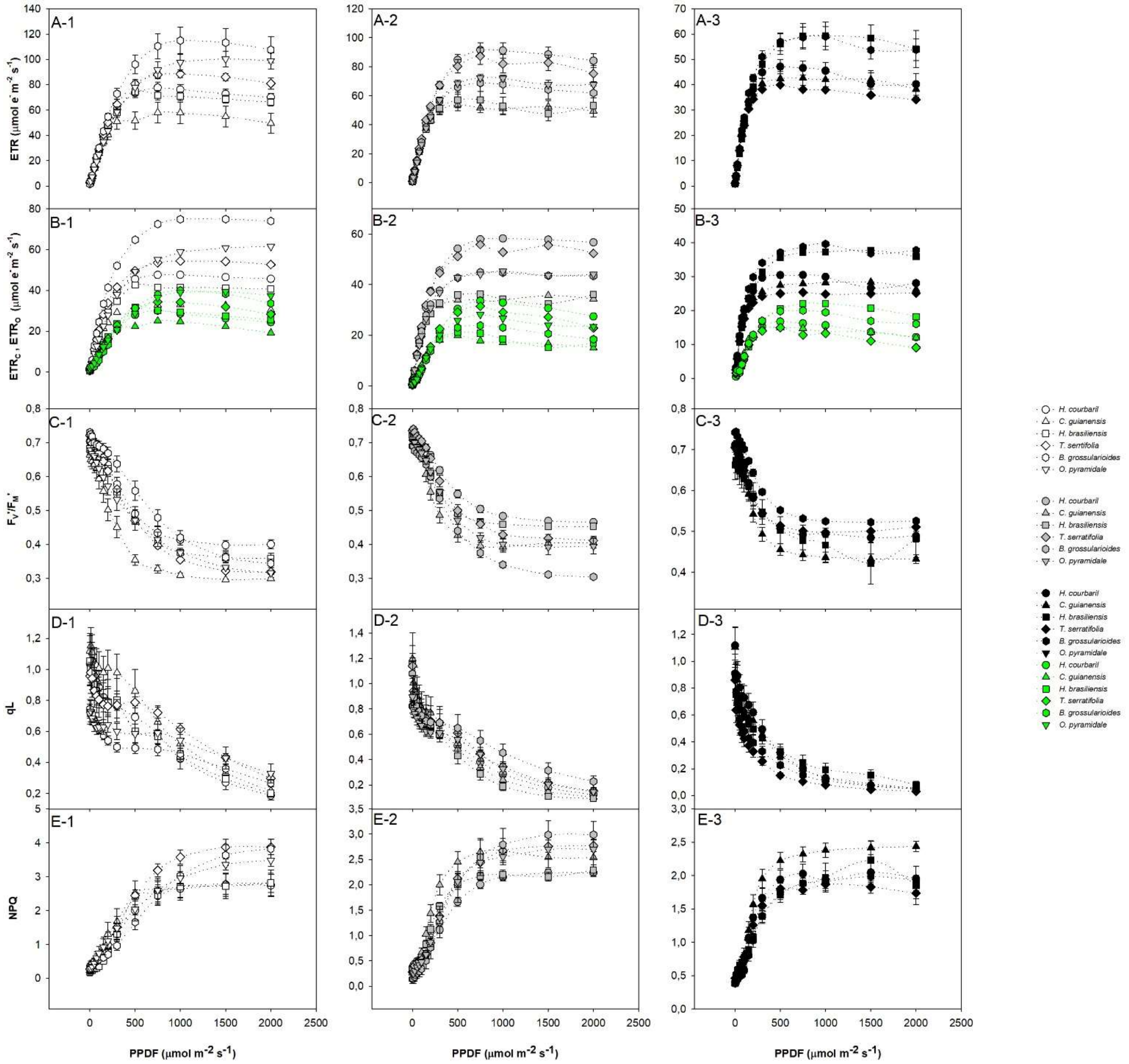

Figure S1: Fluorescence parameters in function of irradiance (PPDF): A) Total electrons transport rate (ETR), B) fraction of electrons destined for carboxylation (ETR_C) and oxygenation (ETR_O – green symbols), C) Maximum efficiency of PSII photochemistry in the light (F_v'/F_m'), D) Photochemical quenching (qL) and E) Non-photochemical quenching (NPQ) of six tree species submitted to three different light environments: full sunlight (open symbols); moderate shade (gray symbols); deep shade (black symbols). Values are mean ± standard error (n = 4).

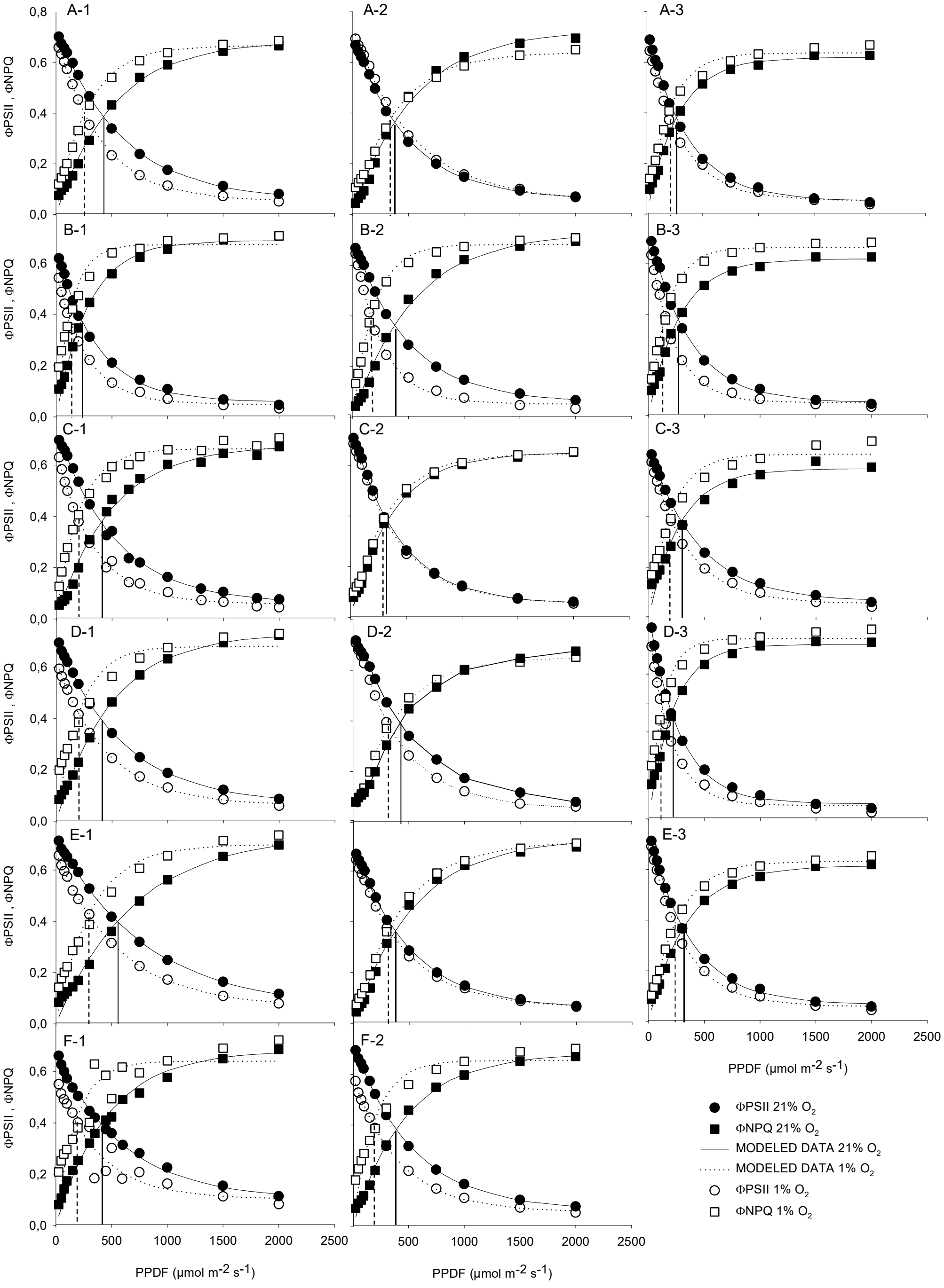

Figure S2: Photochemical and non-photochemical yields of absorbed energy with photosynthetic photon density flux (PPDF) [ΦPSII = m + aexp(–bPPF); ΦNPQ = m(1 – exp(–bPPF));] in seedlings: A) *Hymenea courbaril*, B) *Carapa guianensis*, C) *Hevea brasiliensis*, D) *Tabebuia serratifolia*, E) *Bellucia grossularioides* and F) *Ochroma pyramidale* subjected to three light environments: full sunlight (1); moderate shade (2); deep shade (3) and two O_2 levels. Vertical lines indicate PPF at which ΦPSII = ΦNPQ. Values are mean (n = 4).

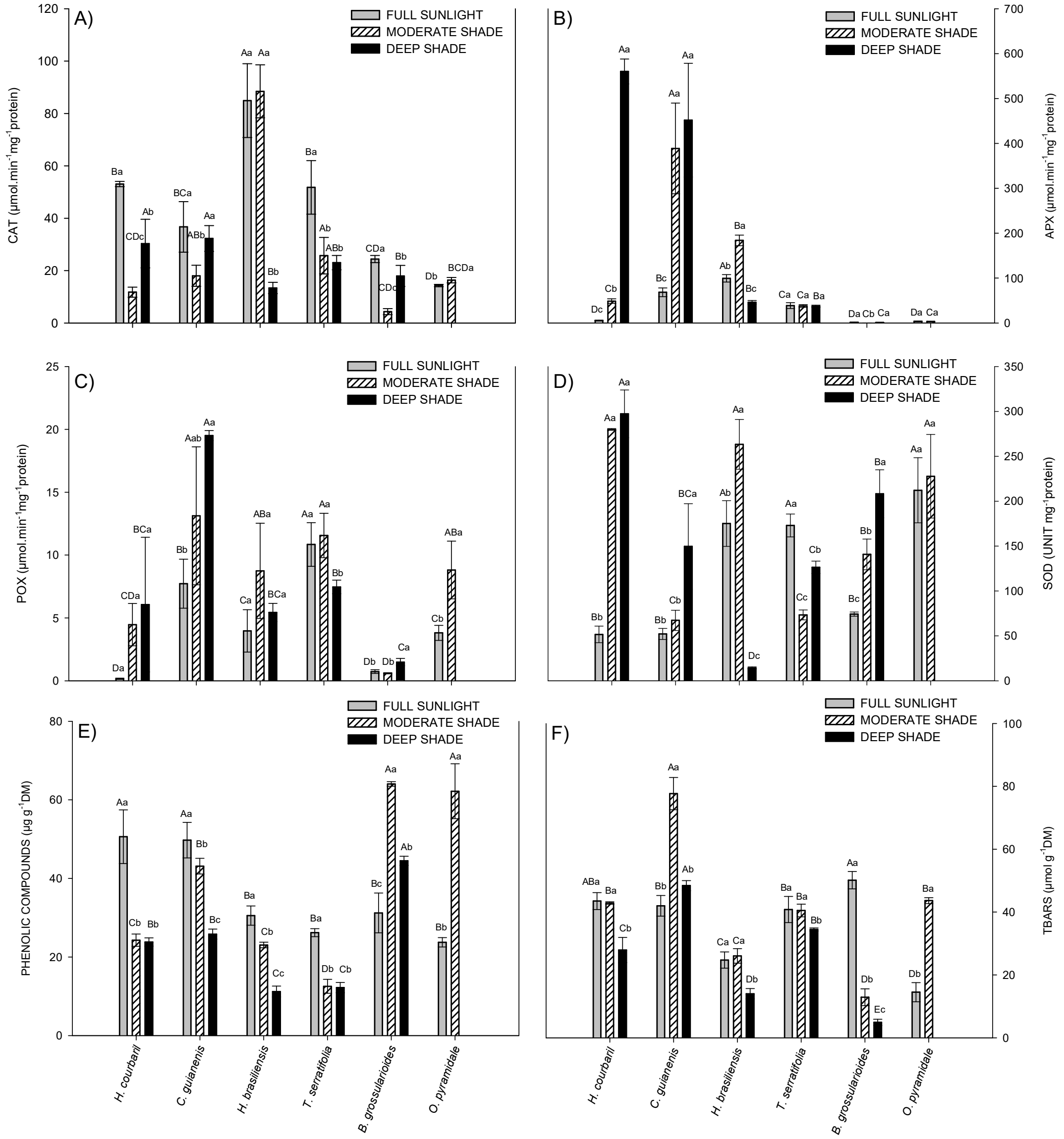

Figure S3: Antioxidant Activity of Enzymes A) Catalase (CAT), B) Ascorbate Peroxidase (APX), C) Phenolic Peroxidase (POX) and D) Superoxide Dismutase (SOD), E) Leaf phenolic compounds and F) Lipid Peroxidation Intensity (TBARS) of six tree species submitted to three different light environments. Same capital letters for different species in same environment and lower case for same species in different environment are equal by Tukey test ($p < 0.05$). Vertical bars indicate the standard error (n=4).

Table S2. Leaf macro and micronutrient content of six tree species submitted to different light environments.

ENVIRONMENT	SPECIE	N ($gkg^{-1}DM$)	P ($gkg^{-1}DM$)	K ($gkg^{-1}DM$)	Ca ($gkg^{-1}DM$)	Mg ($gkg^{-1}DM$)	Fe ($mgkg^{-1}DM$)	Mn ($mgkg^{-1}DM$)	Zn ($mgkg^{-1}DM$)
FULL SUNLIGHT	*H. courbaril*	10.67 ± 0.40BCc	1.04 ± 0.08Cb	2.87 ± 0.23Cb	20.88 ± 6.14 Ba	2.83 ± 0.75ABa	75.25 ± 10.31Bb	145.98 ±39.47 Aa	41.10 ± 9.66Ab
	C. guianensis	9.18 ± 0.28BCb	1.22 ± 0.27 Ca	4.06 ± 1.62BCa	21.30 ± 3.03Bb	3.85 ± 1.14 Aa	49.00 ± 10.80ABb	15.33 ± 1.63CDb	26.43 ± 6.25BCa
	H. brasiliensis	17.01 ± 2.51Ab	1.77 ± 0.39BCa	4.14 ± 1.65BCb	40.45 ± 14.42 Aa	1.13 ± 0.29 Ba	90.00 ± 9.20Ab	47.21 ± 10.28BCa	38.11 ± 5.00ABb
	T. serratifolia	16.63 ± 0.64Ab	1.75 ± 0.08BCa	9.86 ± 0.70 Ac	16.82 ± 5.05 Ba	3.44 ± 1.06 Aa	97.00 ±10.30Ab	69.54 ± 8.31 b	20.51 ± 4.13 b
	B. grossularioides	8.87 ± 0.55Cb	2.72 ± 0.41Ab	6.79 ± 2.14ABb	14.84 ±2.26Bb	3.96 ± 0.48 Aa	82.00 ± 19.13Ab	8.67 ± 0.95Db	31.90 ± 6.58ABCb
	O. pyramidale	12.03 ± 1.73 Ba	2.26 ± 0.57ABb	7.04 ± 2.22ABb	12.70 ± 1.50 Ba	5.06 ± 1.72 Aa	94.25 ± 18.66 Aa	8.70 ± 3.35 Da	11.28 ± 2.92 Da
MODERATE SHADE	*H. courbaril*	15.07 ± 0.39ABb	1.27 ± 0.38 Ba	3.73 ± 0.25Cb	17.10 ± 3.99CDab	2.94 ± 1.39ABa	81.75 ± 10.44CDb	56.35 ± 14.27Ab	76.85 ± 14.99 Aa
	C. guianensis	10.30 ±0.65Bb	1.30 ± 0.26 Ba	4.47 ± 2.30 Ba	32.05 ± 2.35Aab	3.25 ± 0.75ABa	105.75 ± 13.50 Aa	22.70 ± 11.10 Bab	35.33 ±4.10 Ba
	H. brasiliensis	15.51 ± 3.70ABb	2.37 ±1.15 Ba	6.41 ± 1.79Bb	24.55 ± 2.24 Bab	2.42 ± 1.83 Ba	98.00 ± 11.46ABa	43.78 ± 7.02 Aa	21.68 ± 12.19BCb
	T. serratifolia	19.62 ± 5.51Ab	2.14 ± 0.49 Ba	15.93 ± 2.85Ab	6.78 ± 2.29Eb	3.28 ±1.77ABa	61.50 ± 5.32 Da	51.30 ± 10.81Ab	26.73 ± 3.93BCab
	B. grossularioides	9.63 ± 1.40Bb	6.68 ± 2.39 Aa	8.68 ± 0.66Bb	22.53 ± 2.70BCa	5.03 ± 0.63ABa	94.00 ± 10.42BCb	10.20 ± 2.51Bb	39.83 ± 6.18 Bab
	O. pyramidale	14.86 ± 2.91ABa	3.40 ± 0.73 Ba	16.47 ± 3.26 Aa	14.93 ± 2.71 Da	5.63 ± 0.64 Aa	119.50 ± 8.50Ab	17.68 ± 4.30Bb	12.38 ± 1.73 Ca
DEEP SHADE	*H. courbaril*	20.48 ± 3.65 Ba	1.79 ± 0.30BCa	12.46 ± 3.27BCa	11.08 ± 2.19 Bab	4.75 ± 0.99 Aa	127.75 ± 32.50 Ba	46.48 ± 12.22Bb	68.78 ± 9.56ABa
	C. guianensis	15.41 ± 1.74 Ba	1.15 ± 0.30 Ca	6.06 ± 2.45 Ca	28.83 ± 7.83Aab	5.14 ± 2.99 Aa	110.75 ± 19.00 Ba	35.30 ± 9.42 Ba	28.55 ± 5.33 Ca
	H. brasiliensis	26.85 ± 2.93 Aa	2.66 ± 0.36 Aa	11.90 ± 3.64BCa	20.64 ± 7.32ABb	2.83 ± 0.26 Aa	114.00 ± 13.69 Ba	39.33 ± 9.87 Ba	77.78 ± 16.32 Aa
	T. serratifolia	31.98 ± 2.10 Aa	1.73 ± 0.26BCa	27.35 ± 3.72 Aa	21.81 ± 2.62ABa	3.99 ±1.01 Aa	225.00 ± 27.19 Aa	122.15 ± 19.29 Aa	32.38 ± 6.01 Ca
	B. grossularioides	18.63 ± 2.54 Ba	2.21 ± 0.57ABb	15.60 ± 3.94 Ba	19.28 ± 4.34ABab	5.18 ± 0.86 Aa	246.00 ± 51.48 Aa	28.40 ± 2.81 Ba	48.50 ± 3.47BCa
	O. pyramidale								

Mean ± standard deviation (n=4) follow in lines for same capital letter to different species in same environment and lower case to same species in different environment are equal by Tukey test (p< 0,05).
